# Modelling European regional FDI flows using a Bayesian spatial Poisson interaction model

**DOI:** 10.1007/s00168-021-01058-x

**Published:** 2021-04-01

**Authors:** Tamás Krisztin, Philipp Piribauer

**Affiliations:** 1 International Institute for Applied Systems Analysis (IIASA), Schlossplatz 1, 2361 Laxenburg, Austria; 2 Austrian Institute of Economic Research (WIFO), Arsenal 20, 1030 Vienna, Austria

**Keywords:** C11, C21, F23, R11

## Abstract

This paper presents an empirical study of spatial origin and destination effects of European regional FDI dyads. Recent regional studies primarily focus on locational determinants, but ignore bilateral origin- and intervening factors, as well as associated spatial dependence. This paper fills this gap by using observations on interregional FDI flows within a spatially augmented Poisson interaction model. We explicitly distinguish FDI activities between three different stages of the value chain. Our results provide important insights on drivers of regional FDI activities, both from origin and destination perspectives. We moreover show that spatial dependence plays a key role in both dimensions.

## Introduction

Recent decades have shown a rapid growth of worldwide foreign direct investment (FDI), which led to increased efforts in research to understand the economic determinants of FDI activities. Classical explanations focus on the factors driving firms to become multinational. The Ownership-Localization-Internalization theory (see Dunning 2001) explains firms’ motivation as an effort to internalize transaction costs and reap the benefits of externalities stemming from strategic assets.

A large alternative strand of empirical literature builds on trade theory. In this context the drivers of FDI activity are the need for larger sales markets, cheaper source markets, and the willingness to reach a technological frontier (Markusen 1995). Following empirical international economics literature, FDI flows are usually captured within the context of a bilateral spatial interaction model framework. The main advantage of this approach is that it specifically accounts for the role of origin- and destination-specific factors, as well as intervening opportunities. For an overview on the determinants of FDI activities and the location choice of multinationals, see Basile and Kayam (2015), Blonigen and Piger (2014), or Blonigen (2005).

Due to the scarcity of data on FDI activities on a subnational scale, the vast majority of the empirical literature focuses on country-specific FDI patterns. A subnational perspective, however, would allow for in-depth decomposition of the spatial patterns of FDI flows, since FDI sources and destinations are not uniformly distributed within a country, but tend to be spatially clustered. Multiple studies focusing on regional investment decisions of multinational companies (Crescenzi et al. 2013; Ascani et al. 2016a; Krisztin and Piribauer 2020) emphasize within-country heterogeneity of FDI decisions, which can exceed cross-country differences. However, a major gap in the literature is that regional level studies only focus on the destination of FDI decisions, and largely neglect to account for origin-specific factors, as well as intervening opportunities in a subnational context. However, a simultaneous treatment appears particularly important for providing a complete picture on third-regional spatial interrelationships in both source- as well as destination-specific characteristics (Leibrecht and Riedl 2014). Moreover, neglecting to take into account both origin, destination, and third region effects, can lead to biased parameter estimates (Baltagi et al. 2007).

The present paper aims to fill these gaps by focusing on subnational FDI flows in a European multi-regional framework and explicitly accounting for origin-, destination-, as well as third region-specific factors. In this paper, we make use of subnational data from the *fDi Markets* database, which reports on bilateral FDI flows, with detailed information on the source and destination city. This can be compiled to multiple dyadic format, that is each region pair appears twice, corresponding to FDI flowing from one region to the other and vice versa. A specific virtue of the database is that it distinguishes FDI flows by their respective business activity. This allows us to contrast the impact of origin, destination, and third region effects across multiple stages of the global values chain.

Origin- and destination-specific third region effects are captured in our empirical model in two ways. First, the model specification contains spatial contextual effects by means of spatially lagged explanatory variables (see Regelink and Elhorst 2015). Second, we moreover employ an econometric framework in the spirit of Koch and LeSage (2015) and LeSage et al. (2007) which captures spatial dependence using spatially-augmented random effects.

When adopting a subnational perspective, it is crucial to control for spatial dependence, as its presence in regional data is well documented (LeSage and Pace 2009). Even national-level empirical applications clearly document the presence of spatial spillovers on FDI activities. An influential example is the work by Blonigen et al. (2007), who analyse the determinants of US outbound FDI activities in a cross-country framework, while explicitly accounting for spatial dependence among destinations. Further studies which document the presence of spatial issues amongst bilateral (national) FDI activities include Pintar et al. (2016), Regelink and Elhorst (2015), Chou et al. (2011), Garretsen and Peeters (2009), Poelhekke and van der Ploeg (2009), or Baltagi et al. (2007).

We therefore employ a spatial augmented Bayesian Poisson specification on the pan-European subnational level which aims at dealing with both orgin- and destination-specific characteristics. Estimation is achieved using work by Frühwirth-Schnatter et al. (2009), allowing us to deal with high-dimensional specifications in a flexible and computationally efficient way.

The remainder of the paper is organized as follows. Section 2 presents the proposed spatial interaction model, which is augmented by spatial autoregressive origin- and destination-specific random effects, intended to capture spatially dependencies, as well as so-called third region effects. Section 3 details the FDI data, the considered determinants, as well as our selection of regions. In Sect. 4 we assess the determinants of European interregional FDI flows across different stages of the global value chain. The analysis is performed using information on FDI dyads covering 266 NUTS-2 regions in the period 2003–2011. Section 5 concludes.

## A spatial interaction model for subnational FDI flows

This section presents the model specification used for the empirical analysis. It is worth noting that the spatial econometric model is similar to work by LeSage et al. (2007), who aimed at modelling regional knowledge spillovers in Europe. An efficient Bayesian estimation approach for the employed multiplicative form of the Poisson model with spatial random effects is provided in the Appendix.^1^

Let $\varvec{y}$ denote an $N \times 1$ vector containing information on the number of FDI flows between *n* regions.^2^ In the classic spatial interaction model framework the flows are regressed on correspondingly stacked origin-, destination-, and distance-specific explanatory variables, as well as their spatially lagged counterparts. $\varvec{X}_o$ and $\varvec{X}_d$ denote $N \times p_X$ origin- and destination-specific matrices of explanatory variables, respectively. Distances and further intervening factors between the *n* regions are captured by the $N \times p_D$ matrix $\varvec{D}$.^3^ Extending the standard model specification with local spillover effects as well as spatial random effects, we consider a Poisson specification of the form:

2.1 $$\begin{aligned} \varvec{y}&\sim \mathcal {P}(\varvec{\lambda }) \nonumber \\ \varvec{\lambda }&= \exp \left( \alpha _0 + \varvec{X}_o \varvec{\beta }_o + \varvec{X}_d \varvec{\beta }_d + \varvec{D} \varvec{\gamma }_D + \varvec{W}_o \varvec{X}_o \varvec{\delta }_o + \varvec{W}_d \varvec{X}_d \varvec{\delta }_d +\varvec{V}_o\varvec{\theta }_o + \varvec{V}_d\varvec{\theta }_d \right) , \end{aligned}$$

where $\mathcal {P}(\cdot )$ denotes the Poisson distribution and $\alpha _0$ is an intercept parameter. $\varvec{\beta }_o$, $\varvec{\beta }_d$, and $\varvec{\gamma }_D$ are parameter vectors corresponding to $\varvec{X}_o$, $\varvec{X}_d$, and $\varvec{D}$, respectively. The spatial lags of the covariates are captured by $\varvec{W}_o \varvec{X}_o$ and $\varvec{W}_d \varvec{X}_d$, with $\varvec{\delta }_o$ and $\varvec{\delta }_d$ denoting the respective $p_X \times 1$ vectors of parameters. Through these spatial lags we explicitly capture the so-called third region effects (Baltagi et al. 2007), that is origin- and destination-specific spillovers from neighbouring regions. Neighbourhood effects are governed by non-negative, row-stochastic spatial weight matrices, which contain information on the spatial connectivity between the regions under scrutiny. Our Poisson spatial interaction model includes separate spatial weight matrices $\varvec{W}_o$ and $\varvec{W}_d$ to account for origin- and destination-specific third regional effects, respectively.

Origin-based random effects are captured by the term $\varvec{V}_o\varvec{\theta }_o$, where $\varvec{V}_o$ denotes an $N\times n$ matrix of origin-specific dummy variables with a corresponding $n \times 1$ vector $\varvec{\theta }_o$. Similarly, the $n \times 1$ vector $\varvec{\theta }_d$ captures regional effects associated with the destination regions’ matrix of dummy variables $\varvec{V}_d$. We follow work by LeSage et al. (2007) and introduce a further source of spatial dependence via the $n \times 1$ regional effect vectors $\varvec{\theta }_o$ and $\varvec{\theta }_d$, which are assumed to follow a first-order spatial autoregressive process:

2.2 $$\begin{aligned} \varvec{\theta }_o&= \rho _o \varvec{W} \varvec{\theta }_o + \varvec{\nu }_o \varvec{\nu }_o = \mathcal {N}\left( \varvec{0},\phi _o^2 \varvec{I}_n \right) \end{aligned}$$

2.3 $$\begin{aligned} \varvec{\theta }_d&= \rho _d \varvec{W} \varvec{\theta }_d + \varvec{\nu }_d \varvec{\nu }_d = \mathcal {N}\left( \varvec{0},\phi _d^2 \varvec{I}_n \right) , \end{aligned}$$

where $\rho _o$ and $\rho _d$ denote origin- and destination-specific spatial autoregressive (scalar) parameters, respectively. $\varvec{W}$ denotes an $n\times n$ row-stochastic spatial weight matrix with known constants and zeros on the main diagonal.

The disturbance error vectors $\varvec{\nu }_o$ and $\varvec{\nu }_d$ are both assumed to be independently and identically normally distributed, with zero mean and $\phi _o^2$ and $\phi _d^2$ variance, respectively. Note that this assumption implies a one-to-one mapping to origin- and destination-specific normally distributed random effects in the case of $\rho _o = 0$ and $\rho _d = 0$. For a row-stochastic $\varvec{W}$, a sufficient stability condition may be employed by assuming the spatial autoregressive parameters $\rho _o$ and $\rho _d$ to lie in the interval $-1< \rho _o, \rho _d < 1$ (see, for example, LeSage and Pace 2009).

## Bilateral FDI data and regions

Our data set comprises observations on regional FDI dyads for 266 European NUTS-2 regions in the period 2003–2011. A complete list of the regions in our sample is provided in Table 6 in the Appendix.

Observations on regional cross-border greenfield FDI investments stem from the *fDi Markets* database. This database is maintained by fDi Intelligence, which is a specialist division of the Financial Times Ltd. The provided data draws on media and corporate sources to report on the sources and hosts of FDI flows (detailed by country, region, and city), industry classifications, as well as the level of capital investment. Crescenzi et al. (2013) report several robustness tests and detailed comparisons with official data sources. They confirm the reliability of the *fDi Markets* data set, especially with regard to the reported spatial distribution of FDI investments.

Our dependent variables are based on the total amount of inflows from European regions in the period 2003 to 2011. Since the *fDi Markets* data base also contains information on several distinct business activities for both origin and host companies, we follow previous studies by Ascani et al. (2016a) and study the determinants of regional FDI dyads at different stages of the value chain. This information is valuable as investor companies maximize their utility with respect to their position along the value chain. Since specifics of the investor company, as well as details on the FDI investment are largely unobserved, it is crucial to account for the heterogeneity in investor decisions by subdividing industry activities relative to their position along the value chain (see, for example, Ascani et al. 2016a). We therefore define three different classifications: *Upstream*, *Downstream*, and *Production*. The classification adopted in this paper builds on general classifications of the value chain by Sturgeon (2008) and closely tracks the ones employed by Crescenzi et al. (2013) and Ascani et al. (2016a).

Specifically, the upstream category comprises conceptual product development including design and testing, as well as management and business administration activities. The downstream category summarizes consumer-related activities such as sales, product delivery, or support. Finally, the production category includes activities related to physical product creation, including extraction, manufacturing, as well as recycling activities. A complete list of the employed global value chain classification is provided in Table 5 in the Appendix.

Our choices for explanatory variables are motivated by recent literature on (regional) FDI flows as well as regional growth empirics (see, for example, Crespo Cuaresma et al. 2018; Blonigen and Piger 2014; Leibrecht and Riedl 2014; or Blonigen 2005). In most gravity-type models, a region’s ability to emit and attract FDI flows is chiefly captured by its economic characteristics. Our main indicator for economic characteristics is the regions’ market size, proxied by regional gross value added. To control for the degree of urbanization both in origin and host regions, we also include regional population densities as an additional covariate. Empirical evidence suggests (Coughlin et al. 1991; Huber et al. 2017) that higher wages have a deterrent effect on investment. We proxy this in our model by including the average compensation of employees per hour worked as an explanatory variable.

We account for the regional industry mix by including the share of employment in manufacturing and construction (NACE classifications B to F), as well as services (NACE G to U). We moreover include typical supply-side quantities such as regional endowments of human and knowledge capital. To proxy regional human capital endowments, we include two different variables. The first variable measures regional tertiary education attainment shares labelled higher education workers. A second variable labelled lower education workers is proxied by the share of the working age population with lower secondary education levels or less.

We use data on patent numbers to proxy regional knowledge capital endowments. Patent data exhibit particularly desirable characteristics for this purpose, since they can be viewed as a direct result of research and development activities (LeSage and Fischer 2012). In order to construct regional knowledge stocks, we use the perpetual inventory method. We follow Fischer and LeSage (2015) and LeSage and Fischer (2012) to construct knowledge capital stocks $K_{it}$ for region *i* in period *t*. Specifically, we define $K_{it}=(1-r_K)K_{it-1}+P_{it}$, where $r_K=0.10$ denotes a constant depreciation rate and $P_{it}$ denotes the number of patent applications in region *i* at time *t*.

The matrix $\varvec{D}$ includes several different distance metrics. First and foremost, we include the geodesic distance between parent and host regions. Recent empirical literature also consider common language as a potential quantity in $\varvec{D}$ (see Krisztin and Fischer 2015, or Blonigen and Piger 2014). We measure whether the same official language is present in the source and host regions through a dummy variable. Information on official national and minority languages is obtained from the *European Commission*.

Several studies on FDI flows also highlight the importance of corporate tax rates as a potential key quantity to attract FDI inflows (see Blonigen and Piger 2014; Leibrecht and Riedl 2014; Bellak and Leibrecht 2009). Lower corporate income tax rates in the host region as compared to the origin region are thus expected to increase the potential attractiveness of FDI inflows. Matrix $\varvec{D}$ therefore also contains the (country-specific) difference in corporate income tax rates between origin and destination regions. Larger differences are expected to be associated with increasing FDI inflows.

In order to alleviate potential endogeneity problems, we moreover measure all explanatory variables at the beginning of our sample (that is in 2003).^4^ For specification of the spatial weight matrix, we rely on a row-stochastic seven nearest neighbour specification.^5^ Data on the variables used stem from the *fDi Markets*, *Cambridge Econometrics*, as well as the *Eurostat* regional databases. Detailed information on the construction of the dependent and explanatory variables used are presented in Table 1.

**Table 1:** Variables used in the empirical illustration

	Variable	Description
$\varvec{y}$	Upstream	FDI inflows associated with upstream activities. *Source:* *fDi Markets*
	Downstream	FDI inflows associated with downstream activities. *Source:* *fDi Markets*
	Production	FDI inflows associated with production activities. *Source:* *fDi Markets*
$\varvec{X}$	Market size	Proxied by means of regional gross value added, in log terms. *Source: Cambridge Econometrics*
	Population density	Population per square km, in log terms. *Source: Cambridge Econometrics*
	Compensation per hour	Compensation of employees per hours worked, in log terms. *Source: Cambridge Econometrics*
	Employment in industry	Share of NACE B to F (industry and construction) in total employment. *Source: Cambridge Econometrics*
	Employment in services	Share of NACE G to U (services) in total employment. *Source: Cambridge Econometrics*
	Lower education workers	Share of population (aged 25 and over) with lower education (ISCED levels 0-2). *Source: Eurostat*
	Higher education workers	Share of population (aged 25 and over) with higher education (ISCED levels 6+). *Source: Eurostat*
	Regional knowledge capital	Knowledge stock formation measured in terms of patent accumulation, in log terms. *Source: Eurostat*
$\varvec{D}$	Geographic distance	Geodesic distance between source and host region. *Source: Eurostat*
	Difference in tax rates	Country-specific top statutory corporate income tax rates (including surcharges). Measured by means of difference between source and host region. *Source: Eurostat*
	Common language	Dummy variable, 1 denotes that the regions share a common official language, 0 otherwise. *Source: European Commission*

ISCED and NACE refer to the international standard classification of education and the second revision of the statistical classification of economic activities in the European community, respectively

## Empirical results

This subsection presents the empirical results obtained from 15,000 posterior draws after discarding the first 10,000 as burn-ins. Running multiple chains with alternating starting values did not affect the empirical results, which also provides evidence for sampler convergence.

Posterior quantities for upstream-, downstream-, and production-related investment flows are presented in Tables 2, 3, and 4, respectively. Each table reports posterior means and posterior standard deviations for the quantities of interest. Statistical significance of the respective posterior mean estimates is based on a 90% credible interval and highlighted in bold. The first block in each table presents origin- and destination-specific slope parameter estimates, respectively. These estimates are reported for both own region characteristics as well as their spatial lags or third region characteristics (Baltagi et al. 2007). In the spatial econometrics literature, the former are often referred to as average direct impacts. Third region effects captured by spatially lagged counterparts are typically referred to as average indirect (or spillover) impacts (LeSage and Pace 2009). The second block in each table reports posterior summary metrics for the spatial autoregressive origin and destination random effects. The third and last block in each table shows posterior inference for the variables used in the distance matrix $\varvec{D}$.

### Origin- and destination-specific core variables

Table 2 reports posterior parameter estimates for upstream FDI (most notably consisting of business services and headquarters). Starting with the key drivers for regions producing FDI outflows in upstream-related activities, Table 2 shows particularly strong evidence for the importance of the own-regional *market size* and *population density*. In addition, the corresponding third-regional effects are significant and negative. For example, an increase in the market size restricted only to neighbouring regions thus decreases the amount of FDI outflows from a given region. The table also suggests a particularly accentuated importance of a well educated working age population (*higher education workers*) in the origin region. The estimated impact appears much more pronounced as compared to downstream and production FDI. Moreover, for upstream FDI the third region effect associated with the *higher education workers* variable also appears to be positive and highly significant. Own-regional *knowledge capital* endowments appear to be positively associated with the generation of upstream FDI outflows. However, the impacts of *regional knowledge capital* endowments for upstream FDI outflows appear rather muted as compared to the other types of FDI considered. Interestingly, Table 2 shows negative third-regional impacts for *knowledge capital*. Unlike other types of FDI under scrutiny, the *compensation per hour* variable only appears to have a significant impact for own-regional upstream FDI outflows.

Inspection of the regional determinants to attract upstream FDI inflows shows some interesting similarities to the origin-specific characteristics. This holds particularly true for the *market size* and *population density* variables. Both destination-specific variables show a positive and highly significant own-regional impact, with negative (and significant) spatial lags. Similar to the origin specific determinants of upstream FDI, the corresponding host-specific impacts appear more pronounced as in other activity types. This finding is in line with Henderson and Ono (2008), Defever (2006), or Duranton and Puga (2005), who highlight that the location choice of business services and headquarters related activities are particularly driven by functional aspects (rather than by sectoral aspects) and typically tend to be located in urban agglomerations. Regional FDI inflows associated with upstream investment activities moreover appear to be particularly attracted by regions with a higher specialization in the services sector (*employment in services*), relative to the agriculture sector (which serves as the benchmark in the specifications).

**Table 2:** Posterior parameter estimates for FDI associated with upstream value chains.

Variable	Origin		Destination	
	Mean	Std. Dev.	Mean	Std. Dev.
Market size	**1.26**	0.12	**1.32**	0.07
Population density	**0.33**	0.12	**0.29**	0.04
Compensation per hour	**−0.59**	0.31	**−0.94**	0.17
Employment in industry	−1.93	1.31	−0.03	0.75
Employment in services	2.02	1.86	**2.08**	0.83
Lower education workers	−0.01	1.05	−1.03	0.81
Higher education workers	**3.86**	0.66	**4.14**	0.78
Regional knowledge capital	**0.08**	0.03	−0.02	0.05
$\varvec{W}$ Market size	**−2.04**	0.18	**−0.53**	0.10
$\varvec{W}$ Population density	**−0.47**	0.10	**−0.35**	0.06
$\varvec{W}$ Compensation per hour	−0.14	0.26	**−0.63**	0.27
$\varvec{W}$ Employment in industry	−2.04	1.83	0.20	1.31
$\varvec{W}$ Employment in services	1.47	1.57	**−2.79**	1.54
$\varvec{W}$ Lower education workers	0.42	0.93	-0.01	1.01
$\varvec{W}$ Higher education workers	**2.18**	1.01	**2.44**	0.94
$\varvec{W}$ Regional knowledge capital	**−0.94**	0.17	**0.20**	0.10
$\rho _o$, $\rho _d$	**0.58**	0.08	**0.44**	0.09
$\phi _o^2$, $\phi _d^2$	**0.70**	0.08	**1.28**	0.13
Geographic distance	**−1.01**	0.03		
Difference in tax rates	**1.30**	0.63		
Common language	**0.51**	0.07		

The model includes a constant. Results based on 15,000 Markov-chain Monte Carlo iterations, where the first 10,000 were discarded as burn-in. Estimates in bold are statistically significant under a 90% confidence interval

From a theoretical point of view, we would also expect labour costs, measured in terms of *compensation per hours*, to be an important determinant for attracting FDI inflows. This hypothesis is confirmed by inspecting the destination-specific results across all tables. Significant negative direct impacts of this variable can be observed throughout all stages of the value chain, both concerning the own region, as well as third regions. This corroborates the findings of Ascani et al. (2016b), who study the location determinants of Italian multinational enterprises. *Regional knowledge capital* as a pull-factor for upstream FDI inflows appears less relevant. Only the respective third-regional impact is significant, however, it appears comparatively muted.

Overall, the results for downstream FDI reported in Table 3 show a strong similarity to those of upstream FDI (Table 2). This resemblance can be observed for both origin- and destination-specific spatial determinants. For regions as a source of downstream FDI, Table 3 also highlights the key importance of agglomeration forces, proxied by the variables *market size* and *population density*. Both variables show a positive and significant direct impact for the generation of downstream FDI outflows, along with negative third-regional effects. These impacts, however, appear somewhat less pronounced as compared to upstream FDI. Similarly, the impact of regional tertiary education attainment (*higher education workers*) for downstream FDI outflows appears less accentuated as compared to upstream FDI outflows. As opposed to the results for origin-specific upstream FDIs, the third-regional effects of tertiary education attainment are insignificant. *Regional knowledge capital* endowments, on the other hand, appear somewhat more important for generating downstream FDI as compared to upstream FDI, with positive direct, and negative third-regional effects.

**Table 3:** Posterior parameter estimates for FDI associated with downstream value chains.

Variable	Origin		Destination	
	Mean	Std. Dev.	Mean	Std. Dev.
Market size	**0.65**	0.10	**1.26**	0.06
Population density	**0.24**	0.08	**0.17**	0.05
Compensation per hour	−0.43	0.33	**−0.92**	0.17
Employment in industry	−1.33	0.98	**2.38**	1.10
Employment in services	0.61	1.13	**2.97**	0.69
Lower education workers	−1.03	0.74	**−1.10**	0.64
Higher education workers	**2.90**	0.64	**3.29**	0.89
Regional knowledge capital	**0.38**	0.05	**−0.06**	0.03
$\varvec{W}$ Market size	**−1.34**	0.11	**−0.55**	0.15
$\varvec{W}$ Population density	**−0.63**	0.19	−0.13	0.09
$\varvec{W}$ Compensation per hour	**−0.58**	0.21	**−0.48**	0.20
$\varvec{W}$ Employment in industry	**−2.14**	1.13	0.92	0.93
$\varvec{W}$ Employment in services	−1.15	1.68	−1.31	0.98
$\varvec{W}$ Lower education workers	1.01	1.01	**1.53**	0.68
$\varvec{W}$ Higher education workers	1.78	1.04	1.24	0.94
$\varvec{W}$ Regional knowledge capital	**−0.92**	0.17	**0.26**	0.10
$\rho _o$, $\rho _d$	**0.42**	0.12	**0.52**	0.08
$\phi _o^2$, $\phi _d^2$	**0.39**	0.05	**0.75**	0.09
Geographic distance	**−0.85**	0.03		
Difference in tax rates	**3.50**	0.98		
Common language	**0.52**	0.07		

The model includes a constant. Results based on 15,000 Markov-chain Monte Carlo iterations, where the first 10,000 were discarded as burn-in. Estimates in bold are statistically significant under a 90% confidence interval

In line with the prevalent literature (see, among others, Leibrecht and Riedl 2014; Casi and Resmini 2010; or Baltagi et al. 2007), the destination-specific regional determinants for downstream FDI also show a strong importance of the *market size* and *population density* variables as a means to attracting downstream-related FDI inflows. Similar to destination-specific upstream FDI, educational attainment (*lower* and *higher education workers*) and the *compensation per hour* variable appear as important pull-factors. Concerning the regional industry mix, Table 3 suggests that higher shares in the industry and service sectors (*employment in industry* and *services*) appear to be significantly and positively associated with attracting downstream-related FDI inflows. An interesting result is given by a negative and statistically significant own-regional impact of the *regional knowledge capital* variable. The estimated impacts, however, appear rather offset by the positive third-regional impacts. Similar results can also be found in work by Dimitropoulou et al. (2013), a study on the location determinants of FDI for UK regions.

Empirical results for production-related FDI are summarized in Table 4. Starting with the origin-specific determinants of generating production FDI outflows, Table 4 shows not surprisingly a pronounced importance of regional *market size* and *population density*. Similar to the other types of FDI, both variables also exhibit significant negative third-regional effects. Interestingly, the source regional industry mix also appears to play a key role. Specifically, the *employment in industry* variable shows a positive and highly significant direct impact of the origin region. The remaining origin-specific drivers are basically in line with those of the other types of FDI, most notably positive impacts of tertiary education attainment (*higher education workers*) levels and *regional knowledge capital* endowments.

**Table 4:** Posterior parameter estimates for FDI associated with production value chains

Variable	Origin		Destination	
	Mean	Std. Dev.	Mean	Std. Dev.
Market size	**1.01**	0.18	**0.94**	0.09
Population density	**0.14**	0.07	**−0.13**	0.06
Compensation per hour	0.19	0.24	**−1.21**	0.14
Employment in industry	**2.78**	0.80	**4.04**	0.73
Employment in services	−0.07	0.98	**3.10**	0.52
Lower education workers	0.43	0.58	−0.08	0.63
Higher education workers	**2.68**	0.68	0.52	0.62
Regional knowledge capital	**0.20**	0.06	0.00	0.04
$\varvec{W}$ Market size	**−1.11**	0.15	**−0.90**	0.15
$\varvec{W}$ Population density	**−0.41**	0.11	0.02	0.10
$\varvec{W}$ Compensation per hour	−0.38	0.29	−0.61	0.50
$\varvec{W}$ Employment in industry	0.55	1.28	−1.27	1.08
$\varvec{W}$ Employment in services	0.13	1.40	−0.85	0.81
$\varvec{W}$ Lower education workers	**1.04**	0.44	1.14	0.92
$\varvec{W}$ Higher education workers	**2.25**	0.83	**2.03**	0.79
$\varvec{W}$ Regional knowledge capital	**−1.21**	0.14	**0.25**	0.11
$\rho _o$, $\rho _d$	**0.77**	0.05	**0.32**	0.11
$\phi _o^2$, $\phi _d^2$	**0.33**	0.04	**1.05**	0.11
Geographic distance	**−0.96**	0.03		
Difference in tax rates	**1.61**	0.76		
Common language	**0.47**	0.06		

The model includes a constant. Results based on 15,000 Markov-chain Monte Carlo iterations, where the first 10,000 were discarded as burn-in. Estimates in bold are statistically significant under a 90% confidence interval

Inspection of the destination-specific determinants of production-related FDI, however, reveals markedly different patterns as compared to upstream and downstream FDI. Albeit the *market size* shows a similar importance, along with negative third-regional effects, the direct impact of the *population density* variable shows a negative and significant sign. Our estimation results thus show that production-oriented FDI activities are predominantly attracted by smaller regions in proximity to urban agglomerations. For upstream and downstream activities, however, urban agglomerations seem to play a more central role. Moreover, our results imply that regional human capital endowments are particularly important for explaining upstream and downstream-oriented investment decisions. For production activities, the importance of regional human capital endowments appears slightly less pronounced. These results corroborate the findings of Strauss-Kahn and Vives (2009), and Defever (2006) by highlighting that industry-related location decisions typically focus on sectoral, rather than on functional aspects. The significant and positive own-regional, destination-specific industry mix (*employment in industry* and *services*) further underpins these findings.

For attracting production-related FDI, Table 4 shows a particularly pronounced negative impact of the *compensation per hour* variable of the host region. The negative direct impact on inflows is the strongest with a posterior mean of $-1.21$ for production-related activities. However, it is worth noting that the associated third-regional impacts on inflows are insignificant for production, whereas both downstream and upstream related FDI flows exhibit significant negative third-regional impacts. Our findings are moreover in line with Fallon and Cook (2014) and Crescenzi et al. (2013), who both find that locational drivers for production-related FDI flows differ from those associated with business service activities.

**Fig. 1:**
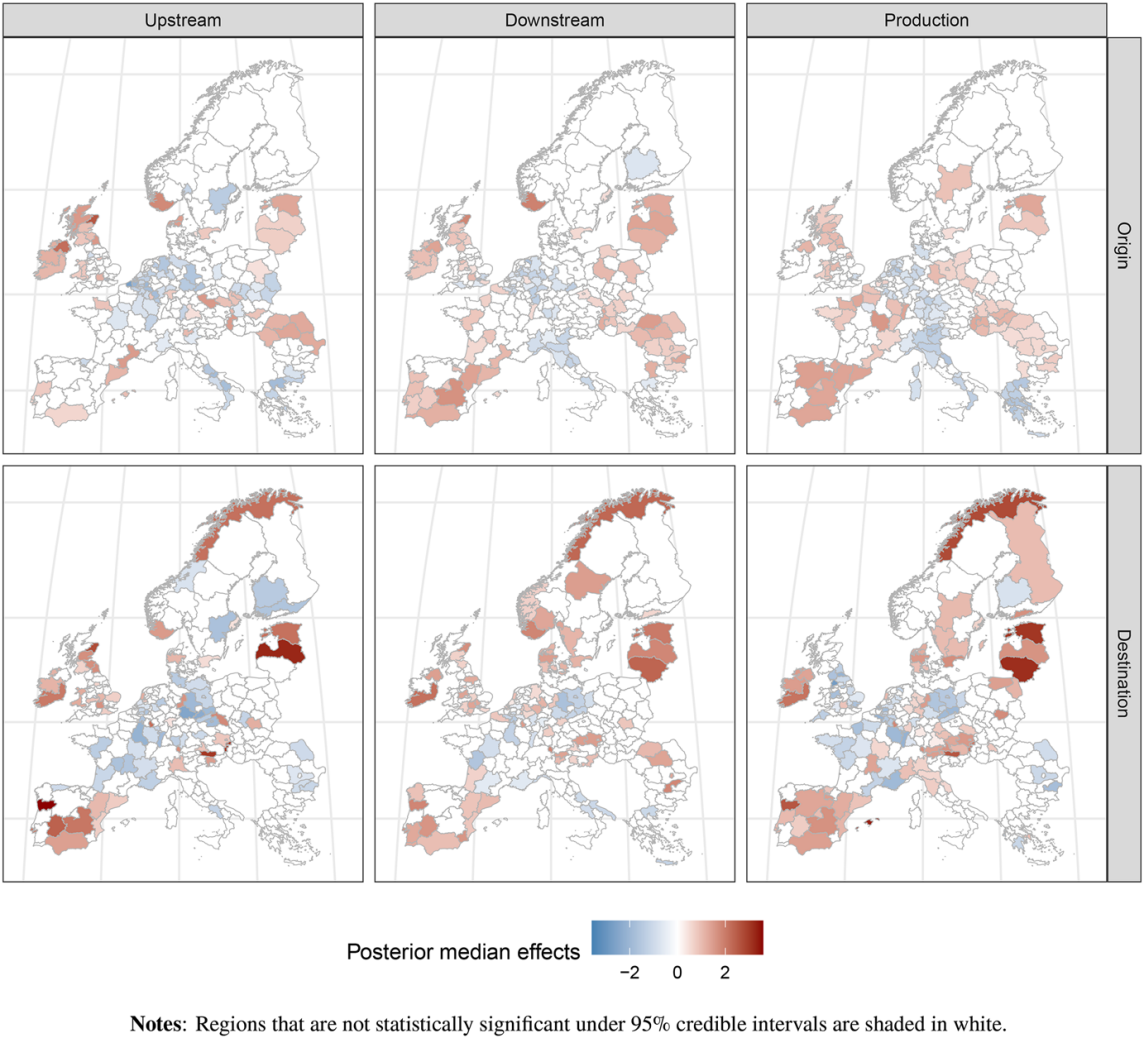
Spatially structured origin- and destination-specific random effects across the value chain. *Notes* Regions that are not statistically significant under $95\%$ credible intervals are shaded in white

### Spatial-dependence and distance metrics

This subsection discusses the results for the spatial autoregressive origin and destination random effects, as well as the estimates of intervening opportunities from the distance matrix $\varvec{D}$. Inspection of posterior estimates for the spatial latent random effects provides significant evidence for pronounced spatial dependence patterns in the random effects across all stages of the value chain. This finding holds true for both source- and host-regional heterogeneity in the sample. Posterior estimates for spatially structured origin- and destination-specific random effects for upstream, downstream and production stages of the value chain are illustrated in Fig. 1. Origin-specific effects are depicted in the top row, while destination-specific effects are in the bottom. Positive values are shaded in red, while negative values are shaded in blue. Regions which were not significant under a $95\%$ posterior credible interval are shaded in white.

A comparison of their corresponding posterior means and standard deviations shows that all spatial autoregressive parameters are estimated with a high precision. The intensity of spatial dependence in the upstream- and downstream-specific latent unobservable effects appear similarly pronounced, with values ranging from 0.42 to 0.58. For production-related investment activities, the difference between $\rho _o$ and $\rho _d$ appears more pronounced, with the former being particularly sizeable (0.77), while the latter appears more muted.

Rather similar results for upstream, downstream and production are also reported for the distance factors collected in matrix $\varvec{D}$. As expected, the posterior mean estimates for *geographical distance* are negative and significantly differ from zero for all types of investment activities. Moreover, the posterior standard deviations are comparatively small, indicating that the impact of *geographic distance* is estimated with a high precision. Higher geographic separation of two regions is thus associated with lower FDI activities, as increased distance often raises transportation, monitoring and thus investment costs. The negative impacts reported in Tables 2, 3, and 4 are in line with recent empirical results in FDI (Leibrecht and Riedl 2014) and trade literature (Krisztin and Fischer 2015).

Our dummy variable measuring whether a pair of regions shares an official *common language* proxies the cultural distance between regions in the sample. As expected, the reported posterior means show a positive sign and are significantly different from zero. The third distance variable in the matrix $\varvec{D}$ measures the (country-specific) difference in *corporate tax rates* between source and target regions. In line with theoretical and empirical literature on the location choice of multinationals, the tables report significant and positive impacts to regional FDI flows when corporate tax rates in the target region are lower than in the source region (see Bellak and Leibrecht 2009 and Strauss-Kahn and Vives 2009). The estimated posterior means for the difference in tax rates suggest that a $1\%$ decrease in the tax rate difference between source and destination regions results in a $1.3\%$ and $3.5\%$ increase in the number of FDI flows for downstream and upstream related activities, respectively.

## Conclusions

This paper presents an empirical study on the spatial determinants of bilateral FDI flows among European regions. Due to data scarcity on the subnational level, previous papers typically adopt a national perspective when analysing FDI dyads (see, for example, Leibrecht and Riedl 2014). This paper thus provides a first spatial econometric analysis on the European regional level by explicitly accounting for origin-, destination-, and third region-specific factors in the analysis. The subnational perspective of our analysis allows us to study the spatial spillover mechanisms of regional FDI flows in more detail. Unlike recent studies on the locational determinants of FDI inflows (see, for example, Ascani et al. 2016b; or Crescenzi et al. 2013), we model FDI decision determinants not only across destination regions but also across the origin regional dimension. Moreover, due to the well-known need to control for spatial dependence when modelling regional data (LeSage and Pace 2009), we also capture spatial dependence through spatially structured random effects associated with origin and destination regions.

Our data comes from the *fDi Markets* database, which contains detailed information on regional FDI activities using media sources and company data. The data from the *fDi Markets* database also contains detailed sectoral information on the functional form of the FDI activity, which allows us to explicitly focus on FDI flows across different stages of the value chain. Specifically, the paper studies the origin- and destination-specific determinants of upstream, downstream, and production activities.

Our empirical results clearly indicate that both source and destination spatial dependence plays a key role for all investment activities under scrutiny. In line with recent literature, we find that regional market size, corporate tax rates, as well as third region effects appear to be of particular importance for all stages in the value chain. We moreover find that production-oriented FDI activities are predominantly attracted by smaller regions in proximity to urban agglomerations. For upstream and downstream activities, however, being in the same region as urban agglomerations seem to play a key role. Moreover, our results imply that regional human capital endowments are particularly important for explaining upstream and downstream-oriented investment decisions. For production activities, the importance of regional human capital endowments are less accentuated. These results corroborate the findings of Strauss-Kahn and Vives (2009), or Defever (2006) by highlighting that industry-related location decisions typically focus on sectoral, rather than on functional aspects. From an origin-specific perspective of FDI activities, our empirical results moreover clearly show that regional knowledge capital endowments appear crucial for host regions to produce FDI outflows. Similar to the results on the destination-specific factors for FDI inflows, we also find high education and agglomeration forces as particularly important aspects for host regional FDI outflows.

## Appendix

**Table 5:** Classification of *fDi Markets* business functions

Classification	Business activities	% of classification
Upstream	Business Services	64.0
	Design, Development and Testing	10.8
	Education and Training	2.5
	Headquarters	12.1
	Information and Communication Technology and Internet Infrastructure	4.3
	Research and Development	6.3
Downstream	Customer Contact Centre	4.2
	Logistics, Distribution and Transportation	26.9
	Maintenance and Servicing	3.4
	Sales, Marketing and Support	62.1
	Shared Services Centre	2.0
	Technical Support Centre	1.4
Production	Construction	21.0
	Electricity	5.3
	Extraction	0.3
	Manufacturing	72.1
	Recycling	1.3

The last column indicates the percent of industry activities per FDI classification. The values are based on the total observed FDI flows in the *fDi Markets* database targeting the selected NUTS-2 regions in the period 2003-2011

**Table 6:** List of regions in the study

Austria	France [continued]	Hungary	Poland [continued]	UK
Burgenland (AT)	Languedoc-Roussillon	Dél-Alföld	Lódzkie	Bedfordshire and Hertfordshire
Kärnten	Limousin	Dél-Dunántúl	Lubelskie	Berkshire, Buckinghamshire and
Niederösterreich	Lorraine	Észak-Alföld	Lubuskie	Oxfordshire
Oberösterreich	Midi-Pyrénées	Észak-Magyarország	Malopolskie	Cheshire
Salzburg	Nord - Pas-de-Calais	Közép-Dunántúl	Mazowieckie	Cornwall and Isles of Scilly
Steiermark	Pays de la Loire	Közép-Magyarország	Opolskie	Cumbria
Tirol	Picardie	Nyugat-Dunántúl	Podkarpackie	Derbyshire and Nottinghamshire
Vorarlberg	Poitou-Charentes	**Ireland**	Podlaskie	Devon
Wien	Provence-Alpes-Côte d’Azur	Border, Midland and Western	Pomorskie	Dorset and Somerset
**Belgium**	Rhône-Alpes	Southern and Eastern	Slaskie	East Anglia
Prov. Antwerpen	**Germany**	**Italy**	Swietokrzyskie	East Wales
Prov. Brabant Wallon	Arnsberg	Abruzzo	Warminsko-Mazurskie	East Yorkshire and
Prov. Hainaut	Berlin	Basilicata	Wielkopolskie	Northern Lincolnshire
Prov. Liège	Brandenburg	Calabria	Zachodniopomorskie	Eastern Scotland
Prov. Limburg (BE)	Braunschweig	Campania	**Portugal**	Essex
Prov. Luxembourg (BE)	Bremen	Emilia-Romagna	Alentejo	Gloucestershire, Wiltshire and
Prov. Namur	Chemnitz	Friuli-Venezia Giulia	Algarve	Bristol
Prov. Oost-Vlaanderen	Darmstadt	Lazio	Àrea Metropolitana de Lisboa	Greater Manchester
Prov. Vlaams-Brabant	Detmold	Liguria	Centro (PT)	Hampshire and Isle of Wight
Prov. West-Vlaanderen	Dresden	Lombardia	Norte	Herefordshire, Worcestershire
Région de Bruxelles-Capitale	Düsseldorf	Marche	**Romania**	and Warwickshire
**Bulgaria**	Freiburg	Molise	Bucuresti - Ilfov	Highlands and Islands
Severen tsentralen	Gießen	Piemonte	Centru	Inner London
Severoiztochen	Hamburg	Provincia Autonoma di Bolzano/	Nord-Est	Kent
Severozapaden	Hannover	Bozen	Nord-Vest	Lancashire
Yugoiztochen	Karlsruhe	Provincia Autonoma di Trento	Sud - Muntenia	Leicestershire, Rutland and
Yugozapaden	Kassel	Puglia	Sud-Est	Northamptonshire
Yuzhen tsentralen	Koblenz	Sardegna	Sud-Vest Oltenia	Lincolnshire
**Czech Republic**	Köln	Sicilia	Vest	Merseyside
Jihovýchod	Leipzig	Toscana	**Slovakia**	North Eastern Scotland
Jihozápad	Lüneburg	Umbria	Bratislavský kraj	North Yorkshire
Moravskoslezsko	Mecklenburg-Vorpommern	Valle d’Aosta/Vallée d’Aoste	Stredné Slovensko	Northern Ireland (UK)
Praha	Mittelfranken	Veneto	Východné Slovensko	Northumberland and Tyne and
Severovýchod	Münster	**Latvia**	Západné Slovensko	Wear
Severozápad	Niederbayern	Latvija	**Slovenia**	Outer London
Strední Cechy	Oberbayern	**Lithuania**	Vzhodna Slovenija	Shropshire and Staffordshire
Strední Morava	Oberfranken	Lietuva	Zahodna Slovenija	South Western Scotland
**Denmark**	Oberpfalz	**Luxemburg**	**Sweden**	South Yorkshire
Hovedstaden	Rheinhessen-Pfalz	Luxemburg	Mellersta Norrland	Surrey, East and West Sussex
Midtjylland	Saarland	**Netherlands**	Norra Mellansverige	Tees Valley and Durham
Nordjylland	Sachsen-Anhalt	Drenthe	Östra Mellansverige	West Midlands
Sjælland	Schleswig-Holstein	Flevoland	Övre Norrland	West Wales and The Valleys
Syddanmark	Schwaben	Friesland (NL)	Småland med öarna	West Yorkshire
**Estonia**	Stuttgart	Gelderland	Stockholm	
Eesti	Thüringen	Groningen	Sydsverige	
**Finland**	Trier	Limburg (NL)	Västsverige	
Åland	Tübingen	Noord-Brabant	**Spain**	
Etelä-Suomi	Unterfranken	Noord-Holland	Andalucía	
Helsinki-Uusimaa	Weser-Ems	Overijssel	Aragón	
Länsi-Suomi	**Greece**	Utrecht	Cantabria	
Pohjois-ja Itä -Suomi	Anatoliki Makedonia, Thraki	Zeeland	Castilla y León	
**France**	Attiki	Zuid-Holland	Castilla-la Mancha	
Alsace	Dytiki Ellada	**Norway**	Cataluña	
Aquitaine	Dytiki Makedonia	Agder og Rogaland	Comunidad de Madrid	
Auvergne	Ionia Nisia	Hedmark og Oppland	Comunidad Foral de Navarra	
Basse-Normandie	Ipeiros	Nord-Norge	Comunidad Valenciana	
Bourgogne	Kentriki Makedonia	Oslo og Akershus	Extremadura	
Bretagne	Kriti	Sør-Østlandet	Galicia	
Centre (FR)	Notio Aigaio	Trøndelag	Illes Balears	
Champagne-Ardenne	Peloponnisos	Vestlandet	La Rioja	
Corsica	Sterea Ellada	**Poland**	País Vasco	
Franche-Comté	Thessalia	Dolnoslaskie	Principado de Asturias	
Haute-Normandie	Voreio Aigaio	Kujawsko-Pomorskie	Región de Murcia	
Île de France				

The not-bold parts are the subnational regions belonging to a country, while the bolded parts are country names

### Detailed description of the Bayesian Markov-chain Monte Carlo algorithm

This section provides a detailed description of the employed Bayesian Markov-chain Monte Carlo (MCMC) algorithm. A similar version is employed by LeSage et al. (2007), who use such a modelling strategy for estimation of knowledge spillovers (measured in terms of patenting dyads) in European regions. Specifically, their estimation approach relies on work by Frühwirth-Schnatter and Wagner (2006), who introduce a Bayesian auxiliary mixture sampling approach for non-Gaussian distributed data. This approach builds on a hierarchical data augmentation procedure by introducing $y_i+1$ latent variables for each observation $y_i$, where $y_i$ denotes the *i*-th element of $\varvec{y}$ (with $i = 1,...,N$).

In order to alleviate the implied computational burden, we rely on an improved version of this auxiliary mixture sampling algorithm (Frühwirth-Schnatter et al. 2013). The algorithm tremendously reduces the number of latent parameters per observation. Specifically, the required number of latent parameters is reduced from $y_i+1$ to at most two per observation for Poisson distributed data (Frühwirth-Schnatter et al. 2013).

From a statistical point of view, $\lambda _i$ from Eq. (2.1) can be interpreted as a parameter in a Poisson process describing occurring events in a given time interval, where $\lambda _i$ denotes the *i*-th element of the Poisson mean $\varvec{\lambda }$. For illustration, imagine sorting all unique values of the observed FDI flows from lowest to highest. The Poisson process can be viewed as modelling – given a specific number of FDI flows – the probability of jumping from one unique value to the next. These two quantities can be characterized as so-called arrival and inter-arrival times. Motivated by this formulation, the distribution itself can be described using merely arrival and inter-arrival times, derived from the rate of the process $\lambda _i$. The expected value of arrival time of $y_i$ is ${1}/{\lambda _i}$ and it follows a Gamma distribution with shape one and rate equal to $y_i$. The inter-arrival times are by definition independent and arise from an exponential distribution with rate equal to $\lambda _i$. Based on this definition, we can model $\lambda _i$ if we sample from the inter-arrival time $\tau _{i1}$ between $y_i$ and $y_i + 1$, as well as for $y_i > 0$, the arrival $\tau _{i2}$ time for $y_i$. The main contribution of Frühwirth-Schnatter et al. (2009) is that they introduce auxiliary variables for $\tau _{i1}$ and $\tau _{i2}$, conditional on $y_i$.

For this purpose let us define the latent variables $\tau _{i1}$ and $\tau _{i2}$, based on the properties of arrival and inter-arrival times:

A.1 $$\begin{aligned} \tau _{i1}&= \frac{\xi _{i1}}{\lambda _i}, \xi _{i1} \sim \mathcal {E}(1) \end{aligned}$$

A.2 $$\begin{aligned} \tau _{i2}&= \frac{\xi _{i2}}{\lambda _i}, \xi _{i1} \sim \mathcal {G}(y_i,1)\quad \forall \,\, y_i > 0, \end{aligned}$$

, where $\mathcal {E}(\cdot )$ denotes the exponential and $\mathcal {G}(\cdot ,\cdot )$ the Gamma distribution. The arrival times $\tau _{i2}$ only apply for $y_i>0$, since zero values have by definition no arrival time. Eqs. (A.1) and (A.2) can be log-linearized in the following fashion:

A.3 $$\begin{aligned} -\ln \tau _{i1}&= \ln \lambda _i + \varepsilon _{i1}, \varepsilon _{i1} = - \ln \xi _{i1} \end{aligned}$$

A.4 $$\begin{aligned} -\ln \tau _{i2}&= \ln \lambda _i + \varepsilon _{i2}, \varepsilon _{i2} = - \ln \xi _{i2}\quad \forall \,\, y_i > 0, \end{aligned}$$

, where for $y_i = 0$ only Eq. (A.3) is defined. Evidently, if $\varepsilon _{i1}$ and $\varepsilon _{i2}$ would be Gaussian this would imply a linear model, which could be easily sampled from. While $\varepsilon _{i1}$ and $\varepsilon _{i2}$ are not Gaussian per se, the distributions can be approximated by a mixture of Gaussians, from which sampling can easily be achieved (Frühwirth-Schnatter et al. 2009).

In order to obtain a model which is conditionally Gaussian, the non-normal density can be approximated by a mixture of $Q(\nu )$ normal components, where $\nu$ denotes the shape parameter of a Gamma distribution. For sampling $\varepsilon _{i1}$ we can set $\nu = 1$, and in the case of sampling $\varepsilon _{i1}$ the rate $\nu$ would be equal to $y_i$. Therefore, the mixture of normal components can be generalised for both distributions. Thus, the mixture distribution is given by the following:

A.5 $$\begin{aligned} p_{\varepsilon }(\varepsilon |\nu ) \sim \sum _{q=1}^{Q(\nu )} w_q(\nu ) \mathcal {N} \left[ \varepsilon | m_q(\nu ), s_q(\nu )\right] , \end{aligned}$$

where $w_q(\nu )$ denotes the weight, $m_q(\nu )$ the mean, and $s_q(\nu )$ the variance. These components, as well as $Q(\nu )$ directly depend on the choice of $\nu$. Values for all these parameters conditional on $\nu$ are provided in Frühwirth-Schnatter et al. (2009). To approximate the Poisson process through the Gaussian mixture in Eq. (A.5), the additional latent discrete variable $\nu _{i1}$, and additionally in cases of $y_i>0$ the discrete variable $\nu _{i2}$ are introduced.

Given $\tau _{i1}$ and $\nu _{i1}$ and additionally for the case of $y_i>0$ $\tau _{i2}$ and $\nu _{i2}$, the conditional posterior of the Poisson model’s slope parameters are Gaussian:

A.6 $$\begin{aligned} -\ln \tau _{i1}&= \ln \lambda _i + m(1) + \varepsilon _{i1},&&\varepsilon _{i1}|\nu _{i1} \sim \mathcal {N}\left[ 0,s(1)\right] \end{aligned}$$

A.7 $$\begin{aligned} -\ln \tau _{i2}&= \ln \lambda _i + m(\nu _{i2}) + \varepsilon _{i2},&&\varepsilon _{i2}|\nu _{i2} \sim \mathcal {N}\left[ 0,s(\nu _{i2})\right]& \forall \,\, y_i > 0. \end{aligned}$$

We can easily sample from the distributions given in Eqs. (A.6) and (A.7) and therefore construct an efficient Gibbs sampling algorithm (for a detailed description, see Section 1 in the Appendix).

For Bayesian estimation, we have to define prior distributions for all parameters in the model. We follow the canonical approach and use a Gaussian prior setup for the parameters $\alpha _0$, $\varvec{\beta }_o$, $\varvec{\beta }_d$, $\varvec{\gamma }_D$, $\varvec{\delta }_o$, and $\varvec{\delta }_d$ with zero mean and a relatively large prior variance of $10^4$. We follow LeSage et al. (2007) in our choice of priors for the spatially structured random effect vectors and set a normal prior structure $\varvec{\theta }_o$ and $\varvec{\theta }_d$, with with zero mean and $\phi _x^2 \left( \varvec{A}_x \varvec{A}_x \right) ^{-1}$ variance, where $x \in [o,d]$ and $\varvec{A}_x = \varvec{I}_n - \rho _x \varvec{W}$. For the variance of the random effects $\phi _x^2$ we employ an inverse Gamma prior with rate equal to 5 and the shape parameter to 0.05. Following LeSage et al. (2007), we elicit a non-informative uniform prior specification $\rho _x \sim \mathcal {U}(-1,1)$.

### The Gibbs sampling scheme

Let us collect the explanatory variables from Eq. (2.1) in an $N \times P$ (with $P = 1 + 4 p_X + p_D$) matrix $\varvec{Z} = [\varvec{\iota }_N, \varvec{X}_o, \varvec{X}_d, \varvec{D},\varvec{W}_o\varvec{X}_o,\varvec{W}_d\varvec{X}_d]$ and $\varvec{\gamma } = [\alpha _0, \varvec{\beta }_o',\varvec{\beta }_d',\varvec{\gamma }_D',\varvec{\delta }_o',\varvec{\delta }_d']'$. Thus, $\varvec{\lambda } = \exp \left( \varvec{Z} \varvec{\gamma } + \varvec{V}_o\varvec{\theta }_o + \varvec{V}_d \varvec{\theta }_d \right)$.

Moreover, let us denote the number of non-zero observations in $\varvec{y}$ as $N_{y>0}$. Then, let $N_+ = N + N_{y>0}$ and let the $N_+ \times 1$ vector $\varvec{y}_+$ be $\varvec{y}_+ = [\varvec{y}', \varvec{y}_{y>0}']'$, where $\varvec{y}_{y>0}$ contains all elements of $\varvec{y}$ which are greater than zero. Moreover, let the $N_+ \times P$ matrix $\varvec{Z}_+$ be $\varvec{Z}_+ = [\varvec{Z}', \varvec{Z}_{y>0}']'$, where the matrix $\varvec{Z}_{y>0}$ contains all rows of $\varvec{Z}$ corresponding to $y_k>0$. In a similar fashion, we augment the dummy observation matrices $\varvec{V}_o$ and $\varvec{V}_d$ and denote the resulting $N_+ \times n$ matrices as $\varvec{V}^+_o$ and $\varvec{V}^+_d$.

Accordingly we order the auxiliary variables corresponding to $\varepsilon _{i1}$ and $\varepsilon _{i2}$ and collect them into the following $N_+ \times 1$ auxiliary variable vectors as $\varvec{\tau } = [\tau _{11} ,..., \tau _{N1},\tau _{12},...,\tau _{N_{y>0}2}]$ and $\varvec{\nu } = [ \nu _{11}, ... , \nu _{N1} , \nu _{12} , ... , \nu _{N_{y>0}2}]$. Based on this, we define the $N_+ \times N_+$ variance matrix $\varvec{\Omega }$. Additionally – based on the definition of the Gaussian mixtures in Eqs. (A.6, A.7) – an $N_+ \times 1$ vector of working responses $\tilde{\varvec{y}}$ can be obtained conditional on $\varvec{\tau }$ and $\varvec{\nu }$, so that $\tilde{\varvec{y}} = m(\varvec{\nu }) - \ln \varvec{\tau }$.

Given appropriate starting values the following Gibbs sampling algorithm can be devised:

I. Sample $\varvec{\gamma }$ from its conditional Gaussian distribution $p(\varvec{\gamma }|\cdot ) \sim \mathcal {N}(\overline{\varvec{\Sigma }}_{\varvec{\gamma }}\overline{\varvec{\mu }}_{\varvec{\gamma }},\overline{\varvec{\Sigma }}_{\varvec{\gamma }})$, where
  $$\begin{aligned} \overline{\varvec{\Sigma }}_{\varvec{\gamma }} = \left( \varvec{Z}_+' \varvec{\Omega }^{-1} \varvec{Z}_+ + \underline{\varvec{\Sigma }}_{\varvec{\gamma }}^{-1} \right) ^{-1} \overline{\varvec{\mu }}_{\varvec{\gamma }} = \varvec{Z}_+' \varvec{\Omega }^{-1} (\tilde{\varvec{y}} - \varvec{V}^+_o \varvec{\theta }_o - \varvec{V}^+_d \varvec{\theta }_d ) + \underline{\varvec{\Sigma }}_{\varvec{\gamma }}^{-1}\underline{\varvec{\mu }}_{\varvec{\gamma }}. \end{aligned}$$
  $\underline{\varvec{\Sigma }}_{\varvec{\gamma }}$ denotes the $P \times P$ prior variance matrix and $\underline{\varvec{\mu }}_{\varvec{\gamma }}$ the $P \times 1$ matrix of prior means.
II. Sample $\varvec{\theta }_x$ from their conditional distributions $p(\varvec{\theta }_x|\cdot ) \sim \mathcal {N}(\overline{\varvec{\Sigma }}_{\varvec{\theta }_x}\overline{\varvec{\mu }}_{\varvec{\theta }_x},\overline{\varvec{\Sigma }}_{\varvec{\theta }_x})$, where
  $$\begin{aligned} \overline{\varvec{\Sigma }}_{\varvec{\theta }_x} = \left( \phi ^{-2}_x {\varvec{A}_x}'\varvec{A}_x + \varvec{V}_x^{+'} \varvec{\Omega }^{-1} \varvec{V}_x^+ \right) ^{-1} \overline{\varvec{\mu }}_{\varvec{\theta }_x} = \varvec{V}_x^{+'} \varvec{\Omega }^{-1} \left( \tilde{\varvec{y}} - \varvec{Z}_+ \varvec{\gamma } - \varvec{V}_x^+ \varvec{\theta }_x \right) . \end{aligned}$$
III. We sample $\phi ^2_x$ from the conditional posterior, which is inverse Gamma distributed and given as $p(\phi ^2_x|\cdot ) \sim \mathcal {IG}(\overline{s}_x,{1}/{\overline{v}_x})$, where
  $$\begin{aligned} \overline{s}_x = (n + \underline{s}_x)/2 \overline{v}_x = \left( \varvec{\theta }_x' \varvec{A}_x' \varvec{A}_x \varvec{\theta }_x + \underline{s}_x\underline{v}_x \right) /2. \end{aligned}$$
  $\underline{s}_x$ and $\underline{v}_x$ denote the prior rate and shape parameters of the inverse gamma distribution $\mathcal {IG}(\cdot ,\cdot )$.
IV. For $\rho _x$ the conditional posterior distribution is:
  $$\begin{aligned} p(\rho _x|\cdot ) \propto |\varvec{A}_x|\exp \left( - \frac{1}{2\phi ^2_x} \varvec{\theta }_x' \varvec{A}_x' \varvec{A}_x \varvec{\theta }_x \right) . \end{aligned}$$
  Unfortunately, this is not a well-known distribution, thus – as is standard in the spatial econometric literature – we resort to a griddy Gibbs step (Ritter and Tanner 1992) in order to sample from the conditional posterior for $\rho _x$.^6^ For this purpose candidate values $\rho _x^*$ are sampled from $\rho _x^* = \mathcal {N}(\rho _x,\pi _{\rho _x})$, where $\pi _{\rho _x}$ is the proposal density variance, which is adaptively adjusted using the procedure from LeSage and Pace (2009) and thus is constrained to a desired interval by the means of rejection sampling. The candidate values are evaluated using their full posterior distributions^7^.
V. For $i=1,...,N$ we sample $\tau _{i1}$ from $\xi _{i1} \sim \text {Ex}(\varvec{\lambda }_i)$ and set $\tau _{i1} = 1 + \xi _{i1}$. If $y_k>0$ then we additionally sample $\tau _{i2}$ from $\mathcal {B}(y_i,1)$ (where $\mathcal {B}(\cdot )$ denotes the Beta distribution) and set $\tau _{i1} = 1- \tau _{i2} + \xi _{i1}$.
VI. For $i=1,...,N$ we sample $\nu _{i1}$ from the discrete distribution involving the mixture of normal distributions with $r=1,...,Q(1)$:
  $$\begin{aligned} p(\nu _{i1} = r|\cdot ) \propto w_r(1) \mathcal {N}\left[ -\ln \tau _{i1}- \ln \lambda _i| m_r(1), s_r(1)\right] \end{aligned}$$
  and for $y_i>1$ we additionally sample $\nu _{i2}$ from the discrete distribution (with $r = 1,...,Q(y_i)$):
  $$\begin{aligned} p(\nu _{i2} = r|\cdot ) \propto w_r(y_i) \mathcal {N}\left[ -\ln \tau _{i2}- \ln \lambda _i| m_r(y_i), s_r(y_i)\right] . \end{aligned}$$
  With the sampled values for $\varvec{\tau }$ and $\varvec{\nu }$, we update $\tilde{\varvec{y}} = \ln \varvec{\tau } - {m}(\varvec{\nu })$ and $\varvec{\Omega }$.

This concludes the Gibbs sampling algorithm. The Markov-chain Monte Carlo algorithm cycles through steps I. to VI. *B* times and excludes the initial $B_0$ draws as burn-ins. Inference regarding the parameters is subsequently conducted using the remaining $B-B_0$ draws.^8^

## Footnotes

1 Detailed R codes for running the proposed model are available upon request.

2 It is worth noting that in the present study *N* is of lower dimension than $n^2$, since FDI dyads by construction exhibit no own-regional and no own-country flows.

3 Detailed information on the straightforward construction of the origin- and destination-specific matrices of explanatory variables $\varvec{X}_o$ and $\varvec{X}_d$ from an $n\times p_X$ dimensional matrix of explanatory variables is provided in LeSage and Pace (2009). LeSage and Pace (2009) also provide detailed guidelines on the convenient construction of origin- and destination-specific spatial weight matrices.

4 To assess the robustness of the results we also estimated a model where the explanatory variables were averages from 2003 to 2011. Overall the estimated quantities and their statistical significance remained unchanged.

5 A series of tests using different number of nearest neighbours for the neighbourhood structure appeared to affect the results in a negligible way.
